# Cardiopulmonary bypass reduces myocardial oxidative stress, inflammation and increases c-kit^+^CD45^−^ cell population in newborns

**DOI:** 10.1186/s12967-018-1478-7

**Published:** 2018-04-27

**Authors:** Johannes Petersen, Andrey Kazakov, Michael Böhm, Hans-Joachim Schäfers, Ulrich Laufs, Hashim Abdul-Khaliq

**Affiliations:** 1 Department of Pediatric Cardiology, Saarland University Medical Center, 66421 Homburg/Saar, Germany; 2 Department of Cardiovascular Surgery, University Heart Center Hamburg, Hamburg, Germany; 3 Department of Cardiology, Angiology and Intensive Care Medicine, Saarland University Medical Center, Homburg/Saar, Germany; 4 Department of Thoracic and Cardiovascular Surgery, Saarland University Medical Center, Homburg/Saar, Germany; 5 Department of Cardiology, University Medical Center Leipzig, Leipzig, Germany

**Keywords:** Cardiopulmonary bypass, Newborns, Congenital heart defects, Myocardial oxidative stress, Inflammation, c-kit^+^CD45^−^ cell

## Abstract

**Background:**

The aim of this study was to characterize the influence of cardiopulmonary bypass (CPB) on myocardial remodeling in newborns and children.

**Methods:**

Biopsies from the right atrium were taken before and after CPB from 4 newborns (5–11 days old) and 7 children (8 months–16 years old). Immunostainings on 10 µm heart tissue frozen sections were performed to detect c-kit^+^ cells, leukocytes (CD45^+^ cells), Ki67^+^ cycling cells. The percentage of 8-hydroxy-guanosine (8-dOHG)^+^cardiomyocytes and non-cardiomyocytes [(8-dOHG)^+^-index] were determined to quantify oxidative stress.

**Results:**

Δ c-kit^+^CD45^−^ cells (resident cardiac stem cells) were increased in newborns (2.2 ± 1.9/mm^2^) and decreased in children − 1.5 ± 0.7/mm^2^, p < 0.01. The (8-dOHG)^+^-index was reduced by 43% in newborns and by 20% in children. CPB did not influence cardiac cell turnover; high cell proliferation was seen in newborns before and after CPB. Cardiopulmonary bypass significantly decreased the leucocyte infiltration in newborns to 40 ± 8%, p < 0.05, but not in children. Infiltration with eosinophils (eosinophils/CD45%) was completely abolished in the myocardium of newborns p < 0.05 and reduced to 22 ± 8% in children after CPB, n.s.

**Conclusions:**

Immediate response and remodeling of the myocardium to CPB differs between newborns, older infants and children. Especially an increased number of c-kit expressing CD45 cells after CPB were seen in neonates in comparison to children. The clinical value of such observation needs to be further assessed in larger cohorts of patients.

**Electronic supplementary material:**

The online version of this article (10.1186/s12967-018-1478-7) contains supplementary material, which is available to authorized users.

## Background

Congenital heart and vessel diseases are the most common organ malformations in newborns [1–3]. According to epidemiological studies from several countries nearly 10 out of 1000 newborns have a congenital heart and/or vessel defect [3–5]. Such congenital heart and vessel defects vary between severe and mild, and the complexity of the cardiovascular defects and their combinations determine the need of immediate therapeutic surgical or catheter interventions. Advances in the diagnostic and therapeutic strategies have improved the immediate and long-term survival and morbidities of these patients [6–8]. Nevertheless, considerable mortality rate in neonates rather than older children in association with corrective cardiac surgery are still challenging. Neonates needing surgical intervention early in life still have significantly higher mortality rates than infants and older children beyond the neonatal period [9, 10]. Corrective cardiac surgery is the most responsible cause for mortality in neonates and infants [11]. However, those neonates and infants have mostly severe congenital heart defects in the need of major palliative or corrective surgical procedures by using extracorporeal cardiopulmonary bypass (CPB). At the moment there is a lack of evidence to propose that delaying surgery may be beneficial for survival. Nevertheless, the morbidities and risk factors associated with CPB, are well documented in adults and children [12, 13]. During corrective surgery cardiac arrest is achieved by perfusion the coronaries with cardioplegic solutions in order to be able to corrective surgical procedures on intra- and extra-cardiac structures. Studies have shown that the duration of cardiac arrest is related to myocardial ischemia and altered myocardial performance after surgery [14–16]. Thus, prevention of myocardium and other organs during corrective surgery is necessary. Further, correct timing of the corrective operation at a newborn age where myocardium is even more vulnerable is important and has to be discussed in regards of the increased risk of myocardial failure after major surgical operations. A few studies considered cell proliferation and the presence of stem cells as residual adaptive mechanisms of the fetal heart against stress and ischemic injury [17–20]. In contrast to the extensive studies on myocardial remodeling in adults with acquired heart disease [21–23] data on the mechanism of adaptation and remodeling in the neonatal heart are still scarce. Therefore, a better understanding of the molecular and genetic mechanisms underlying congenital heart diseases and the specific adapting mechanisms during stress and ischemia is fundamental. Characterization of myocardial injury in standardized myocardial regions before and after CPB may represent the myocardial cell adaptations due to cell injury, apoptotic cell death. Proliferation of stem cells in the injured myocardial tissue may provide information on the adaptive mechanisms and remodeling processes in the neonatal heart. We therefore designed a pilot study and aimed to characterize the myocardial cell changes in myocardial atrial tissues before and after CPB.

## Methods

### Patient characteristics

In this pilot study we included newborns and children who had undergone cardiac surgery in our institution from 2012 until 2013. Written informed consent was obtained from the parents of each study participant. The investigation was approved by the Ethics Committee of Saarland. The study included 4 newborns with different congenital heart diseases [total or partial anomalous pulmonary venous connection (n = 2); hypoplastic left heart syndrome (n = 1); transposition of the great vessels (n = 1)]. Further 7 children were conducted into the study with primarily secundum atrial defect (n = 3), tetralogy of fallot (n = 2), partial atrioventricular defect (n = 1), and subvalvular aortic stenosis (n = 1). Tissue samples from the right atrium of newborns and children were collected before and after CPB from the 4 newborns and 7 children. As proof of concept, myocardial tissue from the left or right ventricle was also taken from 3 of the 7 children during CPB. All newborns were male with a mean age of 7 ± 3 days. 5/7 children were male with a mean age of 1375 ± 2052 days. Intraoperative data showed a significant shorter time of CPB (48 ± 31 vs. 159 ± 54 min; p = 0.002), duration of surgical procedure (121 ± 30 vs. 233 ± 51 min; p = 0.001) as well as duration of hypothermic circulatory arrest (2 ± 6 vs. 15 ± 4 min; p = 0.001) in the children population compared to the newborns. However, aortic cross-clamping time did not differ significantly in both groups (40 ± 14 min vs. 25 ± 15 min; p = 0.153). Further the lowest temperature during surgery was significantly lower in the newborn population compared to the children group (23.3 ± 0.9 min vs. 32.1 ± 4.4 min; p = 0.004).

### Histology and immunofluorescence

Tissue samples from the right atrium and the left ventricle of newborns and children were embedded in tissue freezing medium (Leica Germany) and 10 µm frozen sections were prepared by cryotome (Leica Germany). To detect cardiomyocytes, c-kit^+^ cells, CD45^+^ cells, Ki67^+^ cycling cells and level of oxidative stress immuno stainings on frozen sections were performed by overnight incubation at 4 °C with the primary antibody and incubation with the appropriate secondary antibody at 37 °C for 1 h. Immunofluorescence studies were performed by applying polyclonal antibodies against α-sarcomeric actin (clone5c5, A2172, Sigma-Aldrich, Germany), CD45 (Clones 2B11 and PD 7/26, M0701, DAKO, Germany), c-kit (CD117) (A4502, DAKO, Germany), Ki67 (NCL Ki-67p, Novocastra, UK), 8-hydroxy-guanosine (ab10802, abcam, UK). As secondary antibodies anti-rabbit IgG-TRITC (711-025-152), anti-mouse IgG-TRITC (715-025-150), anti-goat IgG-TRITC (705-025-147) anti-mouse IgM-FITC (115-095-020), anti-rabbit IgG-biotin (711-065-152) and Streptavidin-TRITC (016-020-084) (all Dianova, Germany) were used. The total number of eosinophils was assessed on 10 µm frozen sections stained with hematoxylin–eosin. To perform wash steps phosphate-buffered saline (1× PBS) was used. 4% bovine serum albumin in 1× PBS was used to prevent unspecific binding of antibodies. Sections were counterstained with DAPI (Calbiochem, Germany) and mounted with fluorescent mounting medium (Vectashield, Vector Laboratories, USA) for fluorescence microscopic analysis. All sections were blind and random evaluated using a Nikon E600 epifluorescence microscope (Nikon, Germany) with appropriate filters.

### Apoptosis

Apoptosis detection was performed on 10 µm frozen sections with the ApopTag Peroxidase In Situ Oligo Ligation Kit (Millipore, Germany) according to the manufacture instructions. To evaluate apoptosis rate in cardiomyocytes and non-cardiomyocytes, immunostaining for cardiomyocyte marker α-sarcomeric actin (Sigma-Aldrich, Germany) was performed.

### Tissue morphometry

#### Cardiomyocyte number and myocardial oxidative stress

Numbers of cardiomyocytes and 8-hydroxy-guanosine-positive cells per mm^2^ were determined by examining sections double stained for α-sarcomeric actin and 8-hydroxy-guanosine respectively in at least 15 randomly chosen fields at 1000× magnification (1 section per patient). Nuclei positive for 8-hydroxy-guanosine and α-sarcomeric actin were counted. The cell density was calculated using the formula: cell density (mm^2^) = cell number in 15 fields/((0.00153664) * 15, where 0.00153664 is the area of the sampling grid at 1000× magnification, 15 is the number of counted fields.

#### Cycling Ki67^+^ cells, c-kit^+^ cells, CD45^+^ cells and eosinophils

Morphometric analysis for Ki67^+^, c-kit^+^ and CD45^+^ cells was performed on sections double-stained for Ki67, c-kit, CD45 and α-sarcomeric actin respectively. Total number of eosinophils was assessed on hematoxylin–eosin stained sections. In at least two sections from each patient the total numbers of positive cells were evaluated by screening the total section area. In addition the size of the respective section area was determined by taking pictures at 20× magnification and pooling as well as evaluating them using Lucia G Software. From the total numbers of positive cells per area and from the numbers of cardiomyocytes and non-cardiomyocytes per area the percentages of Ki67^+^ cardiomyocytes, Ki67^+^ non-cardiomyocytes, c-kit^+^ cells, CD45^+^ cells and eosinophils were calculated.

#### Apoptotic cells

For the evaluation of the apoptotic indices, one section from each patient was totally screened for apoptotic cardiomyocytes and non-cardiomyocytes. Apoptosis was detected using light field microscopy for the brown diaminobenzidine staining of the apoptosis kit; the co-immunostaining for the α-sarcomeric actin was evaluated by switching to the fluorescence unit of the same Nikon E600 microscope. After determining the total section area and the numbers of the specific cells as described above, we were able to calculate apoptotic indices as percentage of apoptotic cells in the respective evaluated group.

### Statistical analysis

Results are presented as mean ± standard error of the mean (SEM). Wilcoxon matched pairs test was used to compare parameters before and after cardiopulmonary bypass. Mann–Whitney-test was used for the comparison of two independent groups. For experiments with more than two independent groups, one-way ANOVA with a Fisher LSD post hoc test was used. Correlations were assessed with Spearman analysis. Values of p < 0.05 were considered significant. SPSS version 18.0 (SPSS Inc., Chicago, Illinois) was used for statistical calculations.

## Results

### Cardiopulmonary bypass reduces oxidative stress in the myocardium of newborns

**Fig. 1:**
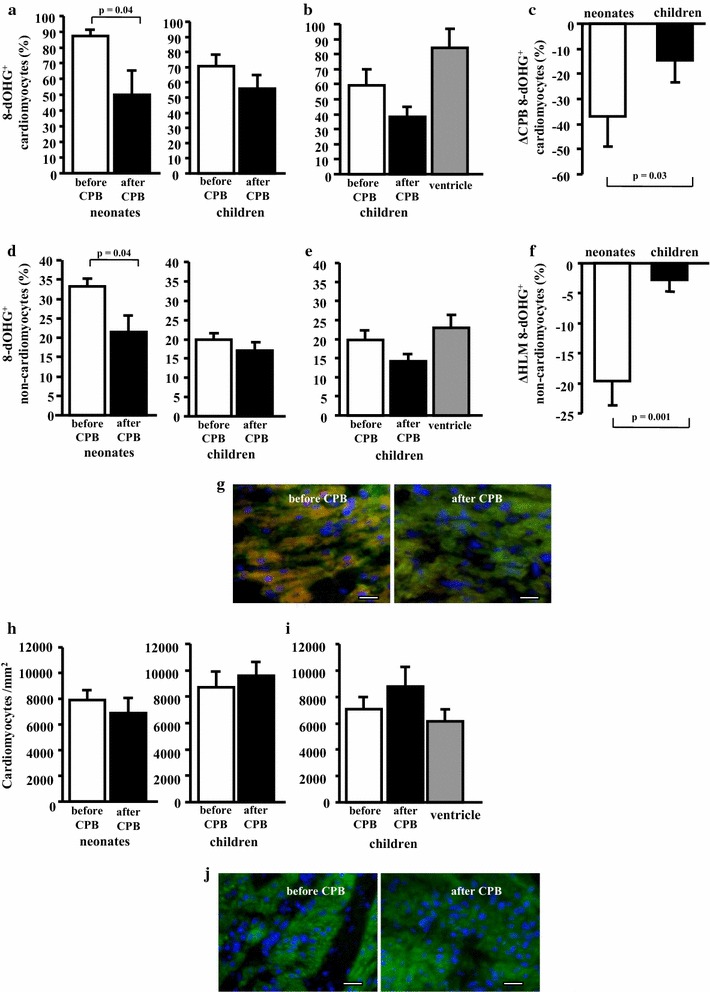
Cardiopulmonary bypass reduces oxidative stress both in cardiomyocytes and in non-cardiomyocytes in newborns. The level of the oxidative stress in the myocardium was assessed by the immunostaining for 8-hydroxy-guanosine (8-dOHG). The percentage of 8-dOHG^+^ cardiomyocytes (**a**) and 8-dOHG^+^ non-cardiomyocytes (**d**) was significantly reduced in the newborns (n = 4) but not in the children (n = 7) after interventions using the heart–lung machine. There was no statistical difference in the percentage of 8-dOHG^+^ cardiomyocytes (**b**) and 8-dOHG^+^ non-cardiomyocytes (**e**) in the biopsies from the atrium and ventricle of children, n = 3 per group. Decrease (ΔCPB) in the percentage of 8-dOHG^+^ cardiomyocytes (**c**) and 8-dOHG^+^ non-cardiomyocytes (**f**) was more pronounced in newborns. Cardiomyocyte density per mm^2^ was not changed after cardiopulmonary bypass both in the newborns (n = 4) and the children (n = 7) (**h**). Cardiomyocyte density was similar in the biopsies from atrium and ventricle of children (n = 3) (**i**). Representative sections from the atrium of a newborn before and after cardiopulmonary bypass demonstrating co-immunostaining for 8-hydroxyguanosine (red) and cardiomyocyte marker alpha-sarcomeric actin (green) (**g**) and immunostaining for alpha-sarcomeric actin (green) (**j**). Nuclei are stained blue by DAPI. Bars = 10 µm

The level of the oxidative stress in cardiomyocytes and in non-cardiomyocytes evaluated by immunostaining for 8-hydroxy-guanosine was significantly reduced after cardiopulmonary bypass in the newborns (8-dOHG^+^ cardiomyocytes before CPB 87 ± 4% vs. after CPB 50 ± 15%, p = 0.04, 8-dOHG^+^ non-cardiomyocytes before CPB 33 ± 2% vs. after CPB 21 ± 4%, p = 0.04) but not in the children (Fig. 1a, c, d, f, g). There was no significant difference in percentage of 8-dOHG^+^ cardiomyocytes and 8-dOHG^+^ non-cardiomyocytes in atrial and ventricular biopsies (Fig. 1b, e). The number of cardiomyocytes per mm^2^ was not changed both in the newborns and children after interventions performed with CPB (Fig. 1h–j).

### Cardiopulmonary bypass does not influence cardiac cell turnover

**Fig. 2:**
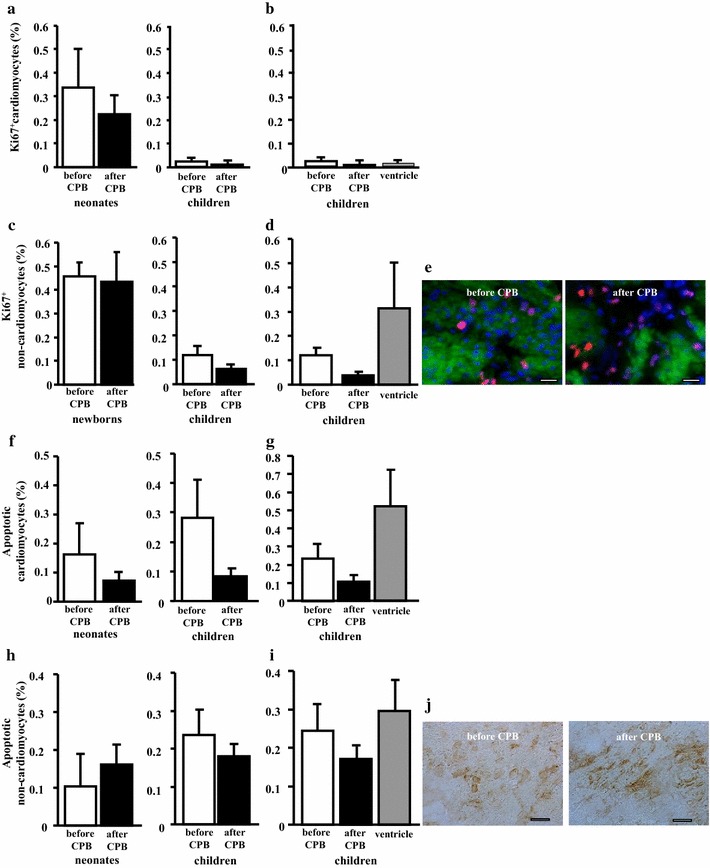
Cardiac cell turnover was not affected by cardiopulmonary bypass. The percentage of Ki67^+^ cycling cardiomyocytes (**a**) and non-cardiomyocytes (**c**) was not affected by cardiopulmonary bypass both in newborns (n = 4) and children (n = 7). The apoptotic rates of cardiomyocytes (**f**) and non-cardiomyocytes (**h**) was similar in both groups before and after cardiopulmonary bypass. There was no statistical difference in the percentage of Ki67^+^ cycling cardiomyocytes (**b**) and non-cardiomyocytes (**d**) and in the percentage of apoptotic cardiomyocytes (**g**) and non-cardiomyocytes (**i**) in biopsies from atrium and ventricle of children (n = 3). Representative sections from the atrium of a newborn before and after cardiopulmonary bypass demonstrating co-immunostaining for proliferation marker Ki67 (red) and cardiomyocyte marker alpha-sarcomeric actin (green) (**e**). Nuclei are stained blue by DAPI. Bars = 10 µm. Representative sections from the atrium of a newborn before and after cardiopulmonary bypass demonstrating light microscopic staining for apoptosis (brown) (**j**). Bars = 30 µm

CPB did not influence cardiac cell turnover. No significant changes were seen in newborns and children before and after CPB. However, a high cell proliferation was seen in newborns before and after CPB in comparison to a low cell proliferation in the children group (Fig. 2a, c, e). The apoptotic rate (Fig. 2f, h, j) of cardiomyocytes and non-cardiomyocytes was not significantly changed after cardiopulmonary bypass both in the newborns and in the children. The difference in proliferation and apoptotic rates of cardiomyocytes and non-cardiomyocytes in atrial and ventricular myocardium was not significant (Fig. 2b, d, g, i).

### Cardiopulmonary bypass reduces inflammatory cell infiltration and increases the number of c-kit^+^CD45^−^ cells in the myocardium of newborns

**Fig. 3:**
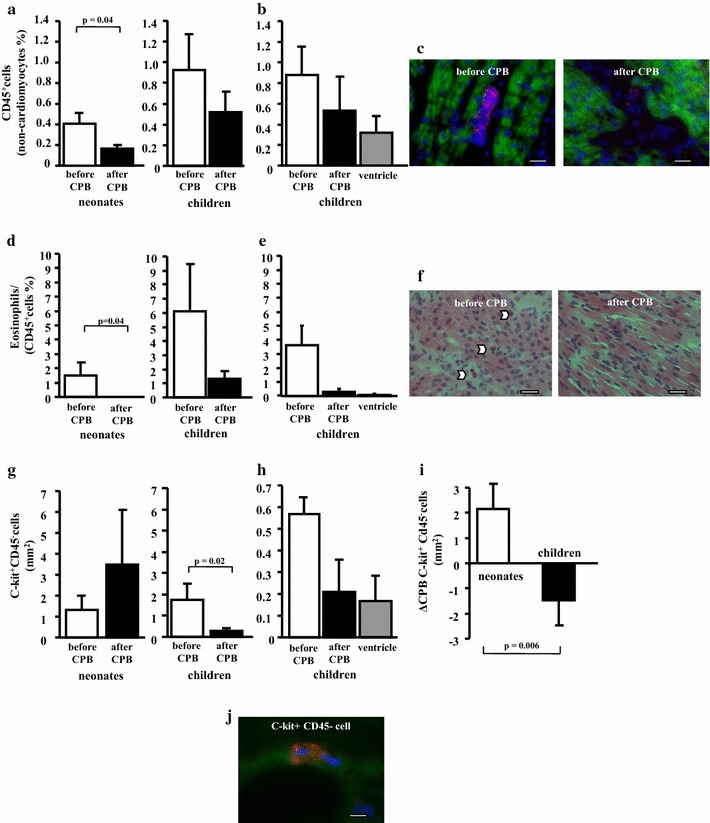
Effects of cardiopulmonary bypass on the inflammatory infiltration and number of c-kit^+^CD45^−^ cells in the myocardial biopsies. Cardiopulmonary bypass significantly decreased the leucocyte infiltration in newborns (n = 4) but not in children (**a**) (n = 7) evaluated by immunostaining for leukocyte common antigen CD45. The percentage of eosinophils was also reduced in newborns after CPB (**d**). In contrast, cardiopulmonary bypass increased the number of c-kit^+^CD45^−^ cells in newborns and decreased their number in children (**g**, **i**). There was no statistical difference in the percentage of CD45^+^cells (**b**), eosinophils (**e**) and in the number of c-kit^+^CD45^−^ cells (**h**) in the biopsies from the atrium and ventricle of children (n = 3). Representative sections from the atrium of a newborn before and after cardiopulmonary bypass demonstrating co-immunostaining for common leucocyte marker CD45 (red) and cardiomyocyte marker alpha-sarcomeric actin (green) (**c**). Nuclei are stained blue by DAPI. Bars = 10 µm. Representative sections from the atrium of a newborn before and after cardiopulmonary bypass demonstrating eosinophils marked by arrowheads. Hematoxylin–eosin staining (**f**). Bars = 10 µm. A c-kit^+^CD45^−^ cell in the atrium of a newborn co-immunostaining for c-kit^+^ CD117 (red) and cardiomyocyte marker alpha-sarcomeric actin (green) (**c**). Nuclei are stained blue by DAPI. Bar = 10 µm

Cardiopulmonary bypass significantly decreased the leucocyte infiltration in newborns assessed by immunostaining for leukocyte common antigen CD45 (before CPB 0.4 ± 0.1% vs. after CPB 0.16 ± 0.03%, p = 0.04) (Fig. 3a, c). Furthermore, infiltration with eosinophils (eosinophils/CD45%) was completely abolished in the myocardium of newborns after cardiopulmonary bypass (before CPB 1.5 ± 0.8% vs. after CPB 0%, p = 0.04) (Fig. 3d, f). In contrast, the newborns demonstrated an increase and the children demonstrated a decrease in the number of c-kit^+^CD45^−^ cells/mm^2^ after cardiopulmonary bypass (ΔCPB c-kit^+^CD45^−^ cells newborns 2.2 ± 2.0/mm^2^ vs. children − 1.5 ± 0.7/mm^2^, p = 0.006) (Fig. 3g, i, j).

## Discussion

The survival of infants and children with congenital heart and vessel defects has improved significantly in the last decades, due to the improvements of diagnostic, surgical, perfusion and myocardial protective strategies [6–8]. However, the increased mortality in those infants requiring surgical therapy in early neonatal life is still challenging. Several studies have shown higher mortality rate in neonates in contrast to older infants and children [12].

Myocardial dysfunction is primarily occurs during the early 24 h post surgery [24]. In addition to the severity of congenital heart defects requiring early surgical intervention, neonatal characteristic structural and functional remodeling mechanisms and higher vulnerability of the neonatal myocardium for ischemia reperfusion injury may contribute to these results [25]. The aim of our pilot study was therefore to characterize remodeling changes in standardized atrial myocardial samples before and after surgical intervention by means of CBP and hypothermic perfusion, which includes setting of myocardial hypoxia.

Recent studies have demonstrated potential endogenic myocardial regeneration under gradual experimental setting of hypoxia [26]. Normal immature myocardium has a greater tolerance to ischemia and fetal and early neonatal heart expressed more adaptation regenerative aspects than adult heart [26]. During the transition from pre- to postnatal life several fetal properties and cell programing in the myocardium may persist for several weeks after birth. Some of these aspects such as cell apoptosis, proliferation, as well as increased number of c-kit^+^CD45^−^ cells which may indicate progenitor stem cell activation in the neonatal myocardium in contrast to the older infants and children, were observed in our present study. The demonstrated differences between neonates and children were also observed before CPB which may confirm the persistent fetal properties in myocardium of neonates rather than in children. The main goal of this study was to evaluate the effect of CPB and possible ischemic events during cardioplegic cardiac arrest on the myocardial cell proliferation, apoptosis and the density of c-kit^+^CD45^−^ cells.

Our results, have demonstrated different responses—and possibly neonatal specific responses—after corrective cardiac surgery using cardioplegic cardiac arrest during CPB. The level of the oxidative stress in both cardiomyocytes and in non-cardiomyocytes was significantly reduced after cardiopulmonary bypass in the newborns in contrast to older children (Fig. 1). Several physiological experimental studies have demonstrated that neonatal myocardium has fewer mitochondria and less oxidative capacity than older children and adults [24, 25]. In contrast, under experimental settings, immature myocardium has a greater tolerance to ischemia, when compared to mature and adult myocardium [26]. In our study we did not find any significant increase of myocardial and non-myocardial cell proliferation immediately after CPB in the neonates. However, the proliferation rate before and after CPB was higher in neonates in comparison to older infants (Fig. 2a, c). The examined tissues were exclusively sampled from the atrial and non-ventricular myocardium. This may explain, in part, the lower level of cell proliferation after CPB in our study. Whether such changes may more detectable in the ventricular myocardium is unclear. Sampling of tissues from the ventricular myocardium before and after CPB under such clinical setting is difficult. However, as proof of concept, we were able to collect ventricular myocardium from 3 children of our study cohort. Those samples were similar in comparison to atrial myocardium.

Significant differences between the neonates and children were found in regard to the effect on the myocardial and non-myocardial cell apoptosis or cell proliferation as studied by means of ApopTag Peroxidase In Situ Oligo Ligation method and immunostaining for proliferation cell marker Ki67. Experimental studies have demonstrated increased number of apoptotic myocardial cells in neonatal in contrast to older lamb [24, 25]. In such experimental setting the intensity of cardioplegic ischemia was significantly higher than the clinical settings, as performed in our studied neonates and infants. In our study, all infants and children survived the operation without significant complications or myocardial dysfunction after corrective heart surgery. Nonetheless, postoperative myocardial stunning and systolic as well as diastolic dysfunction cannot be ruled out with our data. Recently our study group was able to show changes of microRNAs after CPB which are involved in the process of congenital heart defects and myocardial failure [27]. However, no conclusions of the impact of CPB on the neonatal heart can be drawn and further larger studies need to address this question.

Myocardial mitosis and cell proliferation has been considered to be limited to fetal and limited period in the neonatal age [26]. Thus, the adult heart has been considered as a non-regenerative organ after the neonatal age. However, several studies have confirmed myocardial cell proliferation under conditions of ischemia even in adult heart [26]. C-kit^+^ expressing CD45^−^ cardiac cells have been described as multipotent resident cardiac stem cells with capacity to differentiate into endothelial, smooth muscle and myocardial cells [28] and to ameliorate cardiac remodeling [29].

Regarding the number of c-kit^+^CD45^−^ cells after cardiopulmonary bypass, the newborns demonstrated an increase and the children demonstrated a decrease (Fig. 3g, i, j). The presence of such c-kit^+^CD45^−^ cells in our study may present persistent fetal myocardial properties in the newborn in comparison to older children. However, the fact that these c-kit^+^CD45^−^ cells are significantly increased after cardioplegic ischemia during corrective cardiac surgery may confirm potential regenerative capacities in the studied neonates in contrast to older children. The role of c-kit^+^ expressing CD45^−^ cells in the regeneration in response of cardioplegic ischemia is still unclear [28]. In the last years several studies have highlighted the potential regenerative capacities of the neonatal myocardium under ischemic conditions [30]. Neonatal myocardium with increased number of progenitor cells may serve as potential source for autologous transfer of such cells in the future [31]. The first studies in this regard were recently published in small number of infants with hypoplastic left heart syndrome [31–33].

### Study limitations

Our study is limited to descriptive presentation of myocardial regeneration and remodeling after cardioplegic ischemic conditions during corrective cardiac surgery of congenital heart defects. The number of patients was low and the cohort included heterogeneous congenital heart defects which required different surgical corrective modalities. However, this study was designed as a pilot study to asses changes in myocardium after CPB in general. Due to this small and inhomogeneous study population the results are difficult to compare and conclusion can only be drawn carefully. Functional parameter and follow up was not performed since the studied cohort was too heterogeneous.

## Conclusions

Overall this pilot study showed marked differences of myocardial changes after CPB between neonates and children. The data suggest an age specific response to the cardioplegic ischemic conditions during the corrective cardiac surgery using the cardiopulmonary bypass. Especially an increased number of c-kit expressing CD45 cells after CPB was observed in neonates in comparison to children. These results may underlie the value of the regenerative capacities during the neonatal period, which may be in part explained by higher density of progenitor cells in the immature heart. However, due to the small size of the study population no final conclusion can be drawn. Further research will have to assess larger groups of patients and focus on differences between the congenital heart defects.

## Additional file

**Additional file 1.** (1) Information letter to patients and parents, (2) consent form for anonymous publication, participation & storage of medical data and (3) tissue sample form.

## Abbreviations

CPB: cardiopulmonary bypass
8-dOHG: 8-hydroxy-guanosine
(8-dOHG)^+^-index: 8-hydroxy-guanosine^+^cardiomyocytes and non-cardiomyocytes
