# Supplementary figures and images for: Cardiopulmonary bypass reduces myocardial oxidative stress, inflammation and increases c-kit+CD45− cell population in newborns
Source: J Transl Med. 2018 Apr 27;16:111. doi: 10.1186/s12967-018-1478-7 (PMC5921779; doi:10.1186/s12967-018-1478-7)

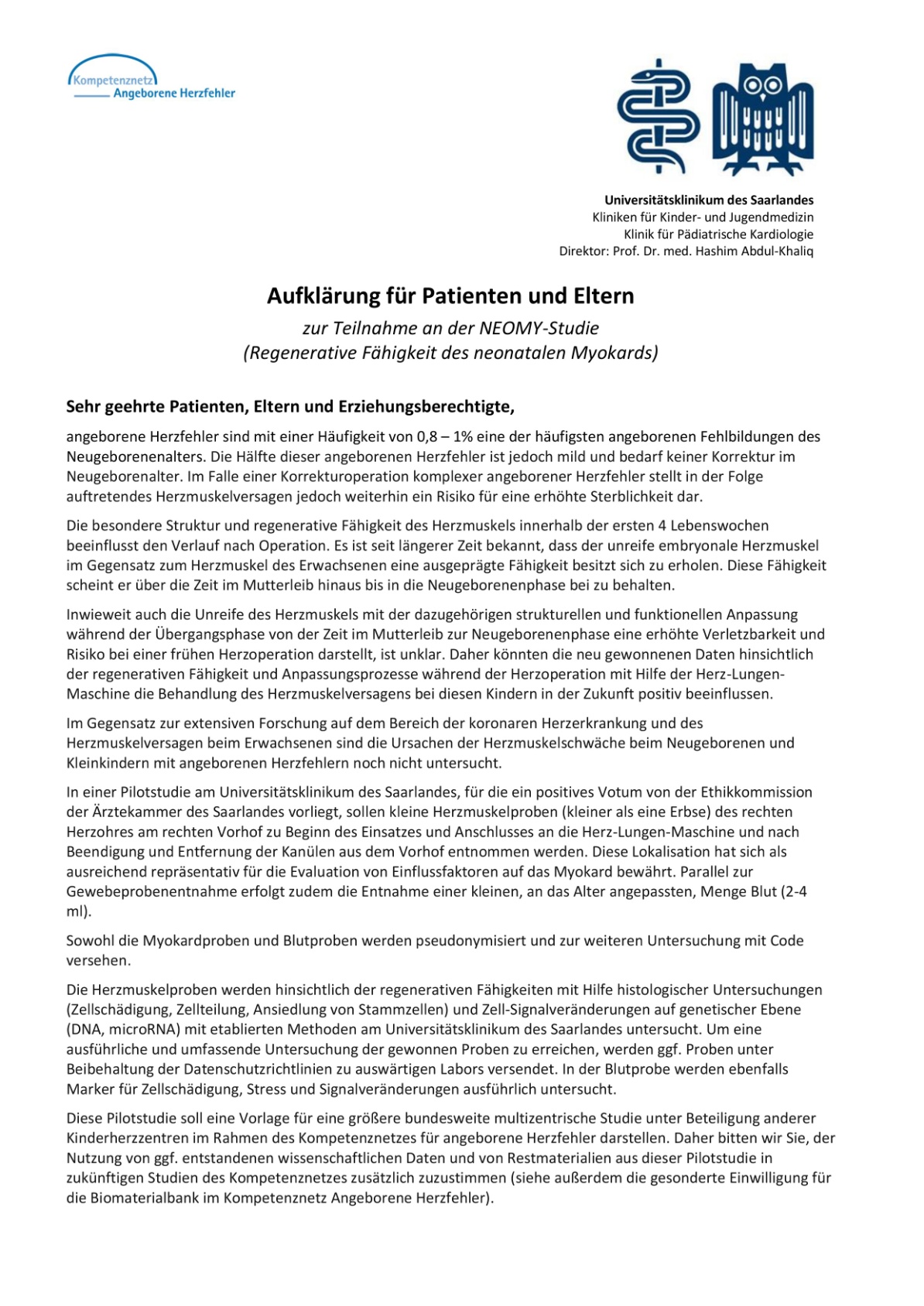


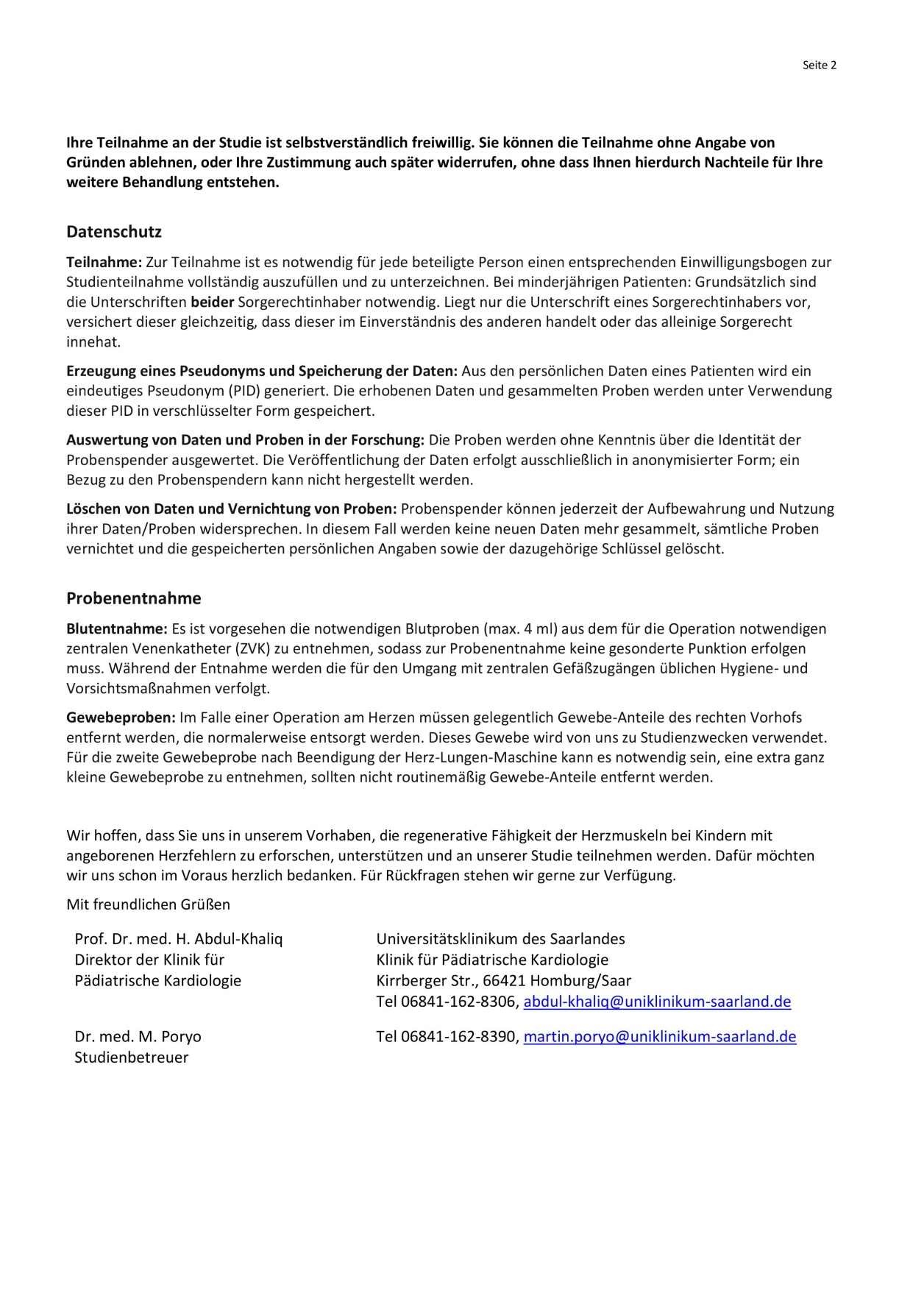


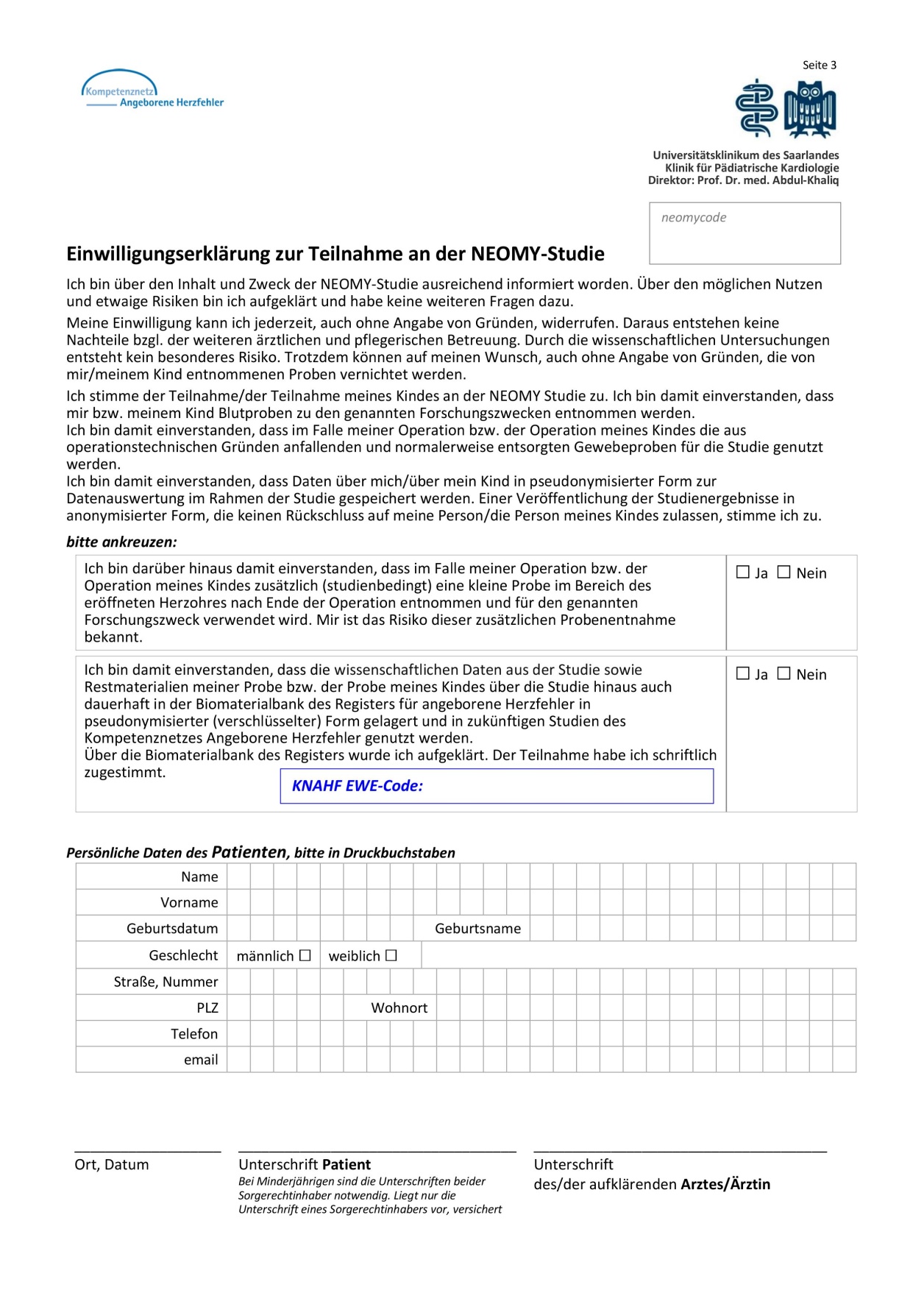


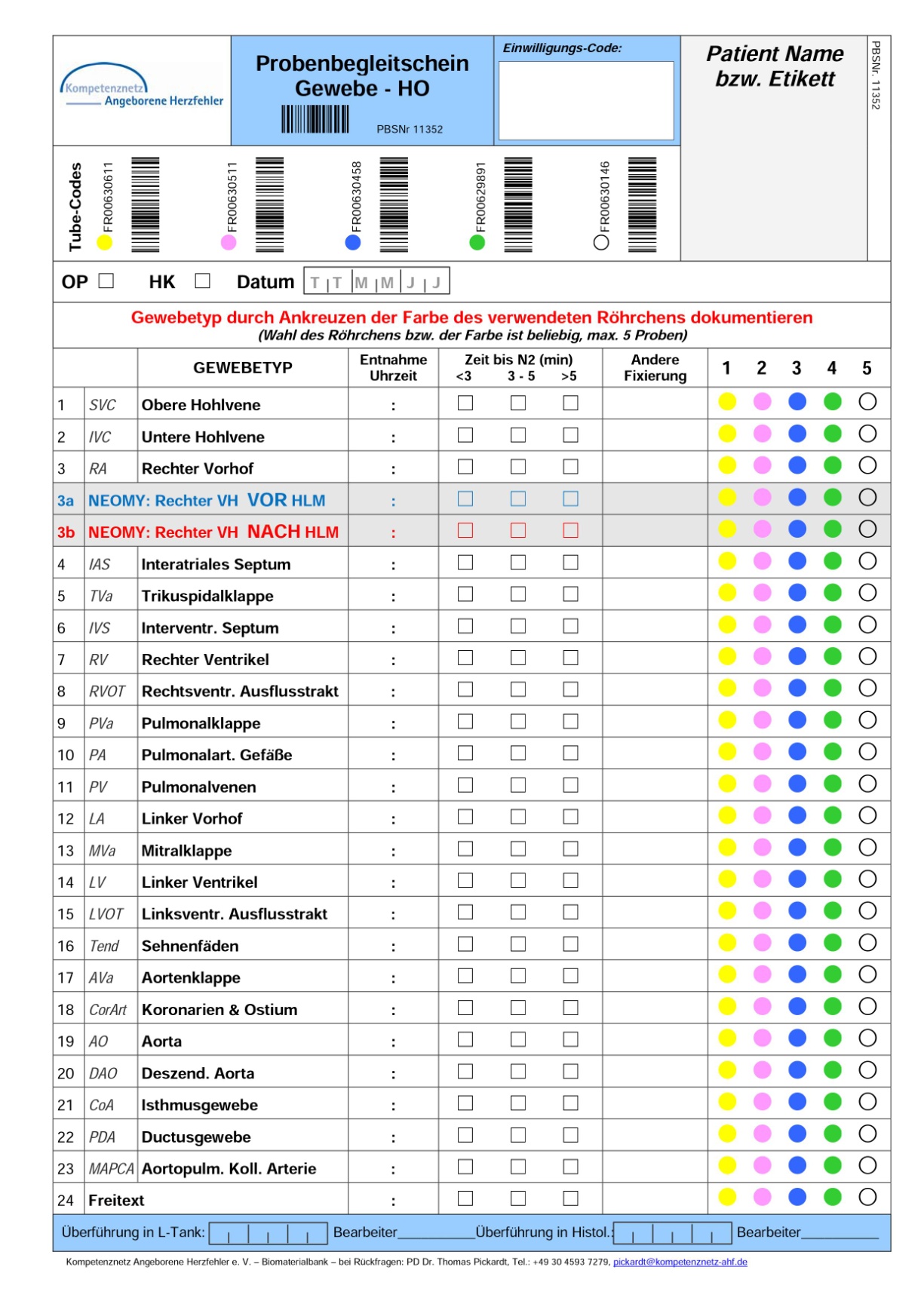

Supplement: Supplementary file 1 — Additional file 1. (1) Information letter to patients and parents, (2) consent form for anonymous publication, participation & storage of medical data and (3) tissue sample form. [file 12967_2018_1478_MOESM1_ESM.docx]
